# Supplementary material for: Dietary Intake and Diet Quality of Adult Survivors of Childhood Cancer and the General Population: Results from the SCCSS-Nutrition Study
Source: Nutrients. 2021 May 22;13(6):1767. doi: 10.3390/nu13061767 (PMC8224580; doi:10.3390/nu13061767)
Supplement: Supplementary file 1 [file nutrients-13-01767-s001.zip › nutrients-1159188-supplementary.pdf]

SUPPLEMENTARY DATA

Supplemental Table S1. Clinical characteristics of childhood cancer survivors

Characteristics	**CCSs (*n* = 775)**
**ICCC-3 diagnosis**, *n (%)*	
I: Leukemia	238 (31)
II: Lymphoma	163 (21)
III: CNS tumor	79 (10)
IV: Neuroblastoma	28 (4)
V: Retinoblastoma	12 (2)
VI: Renal tumor	52 (7)
VII: Hepatic tumor	6 (<1)
VIII: Bone tumor	49 (6)
IX: Soft tissue sarcoma	64 (8)
X: Germ cell tumor	40 (5)
XI & XII: Other tumor	25 (3)
Langerhans cell histiocytosis	19 (2)
**Age at diagnosis**, *n (%)*	
<5 yrs	251 (32)
5-9 yrs	163 (21)
10-14 yrs	225 (29)
15-20 yrs	136 (18)
**Time since diagnosis**,[a] yrs	25.7 (20.0-31.1)
**History of relapse**, *n (%)*	89 (11)
**Treatment exposures**, *n (%)*	
**Radiation**, *n (%)*	
Any	286 (37)
Cranial	120 (15)
<18 Gy	37 (5)
≥18 Gy	83 (11)
Chest	89 (11)
<30 Gy	35 (5)
≥30 Gy	54 (7)
TBI and/or abdominal	68 (9)
**Glucocorticoids**	331 (43)
**Anthracyclines**	293 (38)
**Alkylating agents**	321 (41)
**Hematopoietic stem cell transplantation**	30 (4)

Abbreviations: CCS, childhood cancer survivor; CNS, central nervous system; Gy, gray; ICCC-3, *International Childhood Cancer Classification,* 3rd edition; TBI, total body irradiation.
[a] Values are medians (IQRs).

Supplemental Table S2. Dietary intake in childhood cancer survivors and the general population in Switzerland compared to the DACH dietary recommendations

					Population-based samples			
	DACH recommendations[b]		CCSs (*n* = 775)		Bus Santé[a] (*n* = 2531)		CoLaus[a] (*n* = 1276)	
Nutrients	**men**	**women**	**mean (SD)**	**% DACH[c]**	**mean (SD)**	**% DACH[c]**	**mean (SD)**	**% DACH[c]**
Total energy, kcal[d]	2300-3400	1800-2600	1638 (582)	65	1732 (611)	69	1923 (668)	74
Macronutrients								
Protein, g	57	48	66.1 (29.5)	126	69.8 (28.8)	133	74.8 (29.4)	140
% of energy[e]	10-20	10-20	16.1 (3.6)	108	16.2 (3.6)	108	15.7 (3.3)	104
Vegetable, g			18.1 (7.6)		20.1 (9.2)		22.2 (9.6)	
Animal, g			48.0 (26.9)		49.7 (25.0)		52.6 (24.4)	
Carbohydrates, g[f]	225-275	225-275	180 (74)	72	193 (84)	77	224 (89.9)	90
% of energy[e, f]	45-55	45-55	44.0 (8.8)	88	44.2 (9.1)	88	46.5 (8.1)	93
Mono/disaccharides, g			86 (46)		92 (48)		107 (53)	
Polysaccharides, g			94 (44)		100 (53)		116 (57)	
Total fiber, g[f]	≥30	≥30	12.2 (6.4)	41	14.2 (8.0)	47	15.0 (8.0)	50
Total fat, g			68.1 (28.2)		69.6 (27.5)		73.5 (28.7)	
Total fat, % of energy[e]	30-40	30-40	37.4 (6.9)	107	36.3 (7.0)	104	34.5 (6.2)	99
Saturated fat, g			25.2 (11.6)		24.2 (10.9)		27.5 (12.4)	
Saturated fat, % of energy[g]	<10	<10	13.7 (3.2)	137	12.5 (3.1)	125	12.8 (3.1)	128
Monounsaturated fat, g			28.6 (12.7)		29.5 (12.7)		29.4 (11.9)	
Polyunsaturated fat, g			8.6 (3.8)		9.7 (4.2)		10.3 (4.7)	
Cholesterol, mg[e]	300	300	342 (208)	114	342 (219)	114	323 (141)	108
Micronutrients								
Vitamins								
A, mg-RE[h]	1.0	0.8	0.7 (0.7)	79	0.7 (0.5)	76	0.8 (0.7)	87
D, µg	20	20	2.4 (2.0)	12	3.1 (2.6)	16	2.6 (1.9)	13
Minerals								
Sodium, g[i]	1.5	1.5	2.9 (0.7)	192	2.9 (0.7)	194	3.0 (0.7)	202
Potassium, g	4.0	4.0	2.3 (0.9)	58	2.5 (1.1)	64	2.5 (1.0)	64
Calcium, mg	1000	1000	889 (470)	89	835 (433)	84	1040 (543)	104
Phosphorus, mg	700	700	1178 (451)	168	1194 (473)	171	1234 (476)	176
Iron, mg	104	15	9.0 (3.6)	72	10.1 (3.9)	81	10.8 (3.9)	90
Alcohol, g[i]	20	10	5.7 (7.8)	38	7.9 (10.2)	51	9.3 (12.6)	58

Abbreviations: CCS, childhood cancer survivors; DACH, Dietary recommendations for Germany (D), Austria (A) and Switzerland (CH).
[a] Standardized on sex and age at survey according to CCSs.
[b] DACH recommendations for the general population age 20-50 years, excluding pregnant and lactating women, 2015.

[c] Percentage of mean intake in relation to the DACH recommended intake level * 100. Recommended intake is estimated on the basis of the age-sex groups of the DACH guidelines, weighted by the age and sex distribution of the study population. For alcohol intake the maximum tolerated dosage was taken.
[d] Depending on physical activity level (PAL, 1.4-1.8) and age.
[e] Federal Food Safety and Veterinary Office FSVO, www.naehrwertdaten.ch.
[f] BLV (2009). Kohlenhydrate in unserer Ernährung – Empfehlungen des BLV. www.blv.admin.ch.
[g] BLV (2012). Fett in unserer Ernährung – Empfehlung des BLV. www.blv.admin.chv, recommendations as a percentage of the daily energy demand and based on a daily energy intake of 2000 kcal.
[h] Total vitamin A= retinol + carotene/12, expressed in µg RAE.
[i] Na intake based on FFQ data was calculated with the equation: (8.20 + 0.38 x FFQ in males)/2.54 and (4.55 + 0.67 x FFQ in females)/2.54, with 1 g Na = 2.54 g salt (NaCl) [26]
[j] Maximum tolerated dosage.

Supplemental Table S3. Dietary intake in childhood cancer survivors by sex compared to the DACH dietary recommendations

	DACH recommendations[a]		CCSs (*n* = 775)			
Nutrients	men	women	men (*n* = 386)		women (*n* = 389)	
			mean (SD)	% DACH[c]	mean (SD)	% DACH[c]
Total energy, kcal[b]	2,300-3,400	1,800-2,600	1,743 (632)	61	1,535 (507)	70
**Macronutrients**						
Protein, g	57	48	73.8 (33.8)	129	58.5 (22.0)	122
% of energy[d]	10-20	10-20	16.9 (3.7)	112	15.4 (3.4)	103
Vegetal, g			18.3 (7.7)		17.9 (7.5)	
Animal, g			55.5 (30.7)		40.6 (19.8)	
Carbohydrates, g[e]	225-275	225-275	187 (76.2)	75	174 (71)	70
% of energy[d, e]	45-55	45-55	42.8 (8.4)	86	45.2 (9.1)	90
Mono/disaccharides, g			87 (46)		84 (46)	
Polysaccharides, g			98 (46)		90 (42)	
Total fiber, g[e]	≥30	≥30	11.5 (5.8)	38	12.8 (6.9)	43
Total fat, g			72.2 (30.2)		64.1 (25.4)	
Total fat, % of energy[d]	30-40	30-40	37.2 (6.6)	106	37.5 (7.2)	107
Saturated fat, g			27.3 (12.5)		23.1 (10.4)	
Saturated fat, % of energy[f]	<10	<10	14.0 (2.9)	140	13.5 (3.4)	135
Monounsaturated fat, g			29.9 (13.3)		27.3 (11.9)	
Polyunsaturated fat, g			9.0 (3.9)		8.2 (3.6)	
Cholesterol, mg[d]	300	300	376 (209)	125	310 (202)	103
**Micronutrients**						
Vitamins						
A, mg-RE[g]	0.85	0.7	0.7 (0.7)	71	0.7 (0.7)	89
D, µg	20	20	2.5 (2.0)	12	2.3 (2.0)	12
Minerals						
Sodium, g	1.5	1.5	3.5 (0.1)	236	2.2 (0.2)	149
Potassium, g	4.0	4.0	2.4 (0.9)	61	2.2 (0.9)	55
Calcium, mg	1,000	1,000	908 (502)	91	869 (437)	87
Phosphorus, mg	700	700	1,307 (495)	187	1050 (361)	150
Iron, mg	10[c]	15	9.7 (3.9)	97	8.3 (3.0)	56
Alcohol, g[h]	20	10	7.5 (7.9)	37	4.0 (7.2)	40

Abbreviations: CCS, childhood cancer survivors; DACH, Dietary recommendations for Germany (D), Austria (A) and Switzerland (CH).
[a] DACH recommendations for the general population age 20-50 years, excluding pregnant and lactating women, 2015.
[b] Depending on physical activity level (PAL, 1.4-1.8) and age.
[c] Percentage of mean intake to the DACH recommended intake level * 100. Recommended intake is estimated on the basis of the age-sex groups of the DACH guidelines, weighted by the age and sex distribution of the study population. For alcohol intake the maximum tolerated dosage was taken.
[d] Federal Food Safety and Veterinary Office FSVO, www.naehrwertdaten.ch.
[e] BLV (2009). Kohlenhydrate in unserer Ernährung – Empfehlungen des BLV. www.blv.admin.ch.
[f] BLV (2012). Fett in unserer Ernährung – Empfehlung des BLV. www.blv.admin.chv, recommendations as a percentage of the daily energy demand and based on a daily energy intake of 2000 kcal.
[g] Total vitamin A= retinol + carotene/12, expressed in µg RAE.
[h] Maximum tolerated dosage.

Supplemental Table S4. Scoring method and mean scores (95%CI) of components of the modified Alternate Healthy Eating Index[a, b] by sex in childhood cancer survivors and the general population in Switzerland (Bus Santé and CoLaus)

**Components**					**Population-based samples**			
	**Criteria for**		**Score in CCSs[m] mean (95% CI)**		**Score in Bus Santé[n] mean (95% CI)**		**Score in CoLaus[n] mean (95% CI)**	
	**minimum score (0)**	**maximum score (10)**	**men (*n* =386)**	**women (*n* =389)**	**men (*n* =1,230)**	**women (*n* =1,301)**	**men (*n* =628)**	**women (*n* =648)**
Vegetables, excluding potatoes (servings/day)[c]	0	≥ 5	2.5 (2.3, 2.7)	2.9 (2.7, 3.1)	3.1 (2.9, 3.2)	3.4 (3.3, 3.6)	2.7 (2.5, 2.8)	3.2 (3.0, 3.3)
Fruit, excluding juice (servings/day)[d]	0	≥ 4	2.8 (2.5, 3.1)	3.6 (3.3, 3.9)	3.6 (3.4, 3.7)	3.9 (3.8, 4.1)	3.4 (3.2, 3.7)	4.3 (4.0, 4.5)
Whole grains (g/day)[e]								
Men	0	≥ 90	3.4 (3.1, 3.7)		3.3 (3.1, 3.5)		3.3 (3.0, 3.6)	
Women	0	≥ 75		4.2 (3.9, 4.5)		3.8 (3.6, 4.0)		4.4 (4.1, 4.7)
Sugar-sweetened beverage and fruit juice (servings/day)[f]	≥ 1	0	4.7 (4.4, 5.1)	5.5 (5.1, 5.9)	4.4 (4.2, 4.6)	5.3 (5.0, 5.5)	3.8 (3.4, 4.1)	4.6 (4.3, 5.0)
Nuts, seeds, legumes, and tofu (servings/day)[g]	0	≥ 1	0.7 (0.4, 0.9)	1.0 (0.8, 1.2)	0.8 (0.7, 0.9)	0.9 (0.8, 1.0)	0.5 (0.3, 0.6)	0.7 (0.5, 0.9)
Red and processed meat (servings/day)[h]	≥ 1.5	0	2.8 (2.5, 3.1)	5.6 (5.3, 5.9)	3.7 (3.5, 3.9)	5.5 (5.4, 5.7)	3.5 (3.3, 3.8)	5.3 (5.0, 5.5)
Trans fat (% of total energy intake)[i]	≥ 4	≤ 0.5	9.4 (9.3, 9.4)	9.4 (9.3, 9.4)	9.5 (9.4, 9.5)	9.4 (9.4, 9.4)	9.5 (9.5, 9.6)	9.5 (9.5, 9.5)
Fish, excluding processed products (g/day)	0	32.4	5.9 (5.5, 6.2)	5.7 (5.3, 6.0)	7.7 (7.5, 7.9)	7.4 (7.2, 7.6)	7.7 (7.5, 8.0)	7.6 (7.4, 7.9)
PUFA (% of total energy intake)[j]	≤2	≥10	4.9 (4.6, 5.1)	5.1 (4.9, 5.4)	4.6 (4.4, 4.7)	5.2 (5.1, 5.4)	4.8 (4.6, 5.0)	5.2 (5.0, 5.4)
Sodium (mg/day) [k]	Highest decile	Lowest decile	5.0 (4.7, 5.3)	5.0 (4.6, 5.3)	5.0 (4.9, 5.2)	4.9 (4.8, 5.1)	5.0 (4.7, 5.3)	5.0 (4.7, 5.2)
Alcohol (drinks/day)[l]								
Men	≥ 3.5	0.5 - 2.0	5.7 (5.4, 6.0)		6.1 (5.9, 6.4)		5.9 (5.6, 6.2)	
Women	≥ 2.5	0.5 - 1.5		4.0 (3.6, 4.3)		4.7 (4.5, 4.9)		4.2 (4.0, 4.5)
**Total**	**0**	**110**	**47.7 (46.6, 48.9)**	**51.9 (50.8, 53.0)**	**51.8 (51.1,52.5)**	**54.4 (53.8, 55.1)**	**50.2 (49.3, 51.1)**	**54.0 (53.1, 54.9)**

Abreviations: CCS, childhood cancer survivors; CI, confidence interval; ICCC3, *International Childhood Cancer Classification,* 3rd edition; PUFA, polyunsaturated fatty acids.

[a] Adapted from Chiuve et al. J Nutr 2012 142(6):1009-18

[b] Intermediate food intake was scored proportionately between the minimum score 0 and the maximum score 10.

[c] One serving was equal to 118.3g of raw or cooked vegetables or 250g of vegetable soup. Dried vegetables were not included.

[d] One serving was equal to 118.3g of raw or cooked fruit. Dried fruit was not included.

[e] Whole grain products like whole grain bread and muesli were included.

[f] One serving was equal to 226.8g.

[g] One serving was equal to 28.4g.

[h] One serving was equal to 113.4g of red meat or 42.5g of processed meat.

[i] Consumption was estimated based on the assumption that each food contains a maximum of 2g of trans fat per 100g of total fat, as defined in Swiss regulation.

[j] The highest score was given to individuals with ≥10% of total energy intake from polyunsaturated fatty acids (PUFA).

[k] Na intake based on FFQ data was calculated with the equation: (8.20 + 0.38 x FFQ in males)/2.54 and (4.55 + 0.67 x FFQ in females)/2.54, with 1 g Na = 2.54 g salt (NaCl) [26]. Within CCSs mean values in lowest decile were 2.0g/d in women and 3.4g/d in men and in highest decile were 2.7g/d in women and 3.9g/d in men; Within Bus Santé mean values in lowest decile were 2.0g/d in women and 3.4g/d in men and in highest decile were 2.7g/d in women and 3.9g/d in men; Within CoLaus mean values in lowest decile were 2.0g/d in women and 3.4g/d in men and in highest decile were 2.8g/d in women and 3.8g/d in men

[l] One drink was 113.4g of wine, 340.2g of beer or 42.5g of liquor. A score of 2.5 was given to nondrinkers.

[m] Adjusted for sex, age at survey, and ICCC-3 diagnosis
[n] Adjusted for sex and age at survey and standardized on sex and age at survey according to CCSs.

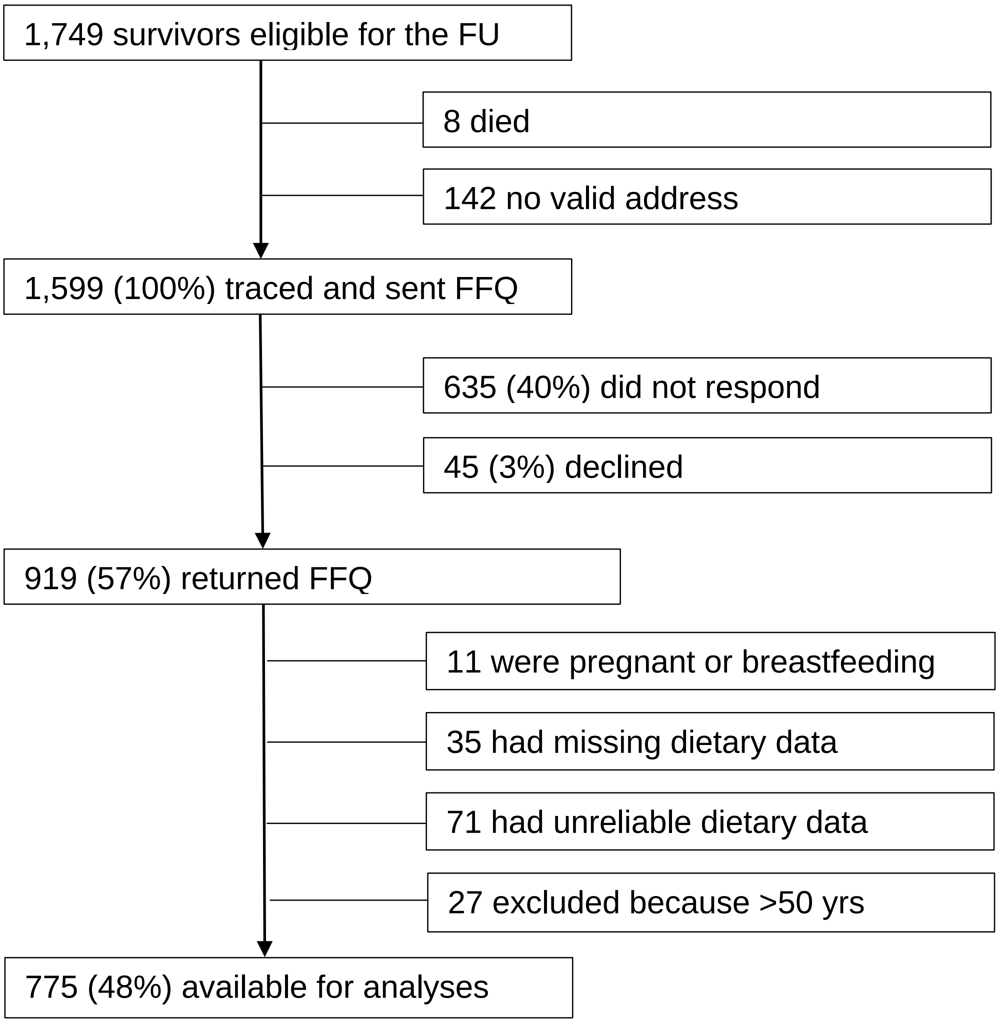

Supplemental Figure 1. Response rates in the Swiss Childhood Cancer Survivor Study (SCCSS)-Nutrition study

Abbreviations: FFQ, food frequency questionnaire; FU, follow-up;

ALIMENTATION

1. Suivez-vous un régime alimentaire?

Non ☐
Oui ☐

Si oui, lequel?

2. Lequel des aliments suivants **excluez-vous totalement** de votre alimentation? ***(plusieurs choix possible)***

œufs ☐
viande (bœuf, porc, veau, etc.) ☐
volaille (poulet, dinde, canard etc.) ☐
fruits ☐
légumes ☐
poissons ☐
fruits de mer ☐
fromages ☐
lait ☐
autres produits laitiers ☐
fruits à coque ☐
céréales ☐
alcool ☐
aucun des aliments ci-dessus ☐

Instructions pour remplir le questionnaire

Par ce questionnaire, nous souhaitons connaître votre alimentation au cours des **4 dernières semaines.**

Pour chaque type d'aliment, nous vous demandons de **comparer votre portion habituelle à la portion de référence**, selon l'exemple ci-dessous.

Exemple
Cette personne a mangé le mois dernier du pain complet midi et soir, donc 2 fois par jour; elle a mangé 2 tranches de pain à chaque fois, donc plus que la portion de référence.
Elle a mangé du beefsteak 1 à 2 fois par semaine, sa tranche de viande pesant environ 150 grammes donc pareille à la portion moyenne de référence.

ALIMENTS	FREQUENCE							QUANTITE			
	Jamais ces 4 dernières semaines	1 x par mois	2 à 3 x par mois	1 à 2 x par semaine	3 à 4 x par semaine	1 x par jour	2 x ou plus par jour	Portion de référence	Votre portion		
									moins	pareil	plus
Pain complet, au seigle							X	50g = 1 tranche			X
Beefsteak, cheval, veau (escalope, filet)				X				150g		X	

Avec les questions suivantes, nous aimerions savoir votre alimentation au cours **des 4 dernières semaines.**

ALIMENTS		FREQUENCE							QUANTITE			
		Jamais ces 4 dernières semaines	1 x par mois	2 à 3 x par mois	1 à 2 x par semaine	3 à 4 x par semaine	1 x par jour	2 x ou plus par jour	Portion de référence	Votre portion		
										moins	pareil	plus
**Fromages, Yaourts**												
1	yaourt nature								1 yaourt = 180g			
2	yaourt light, flan light, crème light								1 yaourt = 180g			
3	yaourt aux fruits, flan, crème dessert								1 yaourt = 180g			
4	fromage blanc 0%								120g			
5	fromage blanc 20%, ricotta, séré, cottage-cheese								100g			
6	feta, mozzarella, fromage frais ½ sel, petit-suisse								40g = 1 petit-suisse			
7	gruyère, tomme, camembert, bleu, parmesan								40g = taille d'une boîte d'allumettes			
8	fondue au fromage								20 bouchées = 3 à 4 tranches de pain			
**Pain, Céréales**												
9	pain blanc, pain de mie, de campagne, au lait, tresse								50g = 1 tranche			
10	pain complet, pain au seigle								50g = 1 tranche			
11	müesli ou autres céréales mélangées								50g = ½ gobelet de yaourt			
12	corn-flakes, blé soufflé, céréales soufflées								30g = 1 gobelet de yaourt			
13	biscottes, cracottes, pain grillé «suédois»								18g = 3 pièces			

2

ALIMENTS		FREQUENCE							QUANTITE			
		Jamais ces 4 dernières semaines	1 x par mois	2 à 3 x par mois	1 à 2 x par semaine	3 à 4 x par semaine	1 x par jour	2 x ou plus par jour	Portion de référence	Votre portion		
										moins	pareil	plus
**Viandes**												
14	beefsteak, cheval veau (escalope, filet)								150g			
15	poulet sans peau								80g = 1 blanc de poulet			
16	poulet avec peau								80g = 1 blanc de poulet			
17	steak haché, entrecôte, rôti (bœuf, porc, veau)								100g			
18	jambon, pot-au-feu, côtelettes d'agneau, côtes de porc								50g = 2 tranches fines de jambon ou 1 côtelette			
19	saucisson, jambon cru, lard maigre, salami								50g = 2 tranches			
20	pâté, terrine								50g = ½ tranche			
21	cervelas								1 pièce			
22	saucisse								1 pièce			
23	foie de veau, de génisse, de porc								130g = 1 tranche			
24	foie de volaille								50g = 1 pièce			
**Poissons, Crustacés**												
25	saumon frais, saumon fumé								100g = 1 darne = 2 tranches			
26	poisson frit, pané, filets de perche								150g = 3 grand sticks			
27	thon à l'huile								40g = ½ boîte			
28	poisson maigre type cabillaud, colin, truite								150g			
29	crevettes, coquillages								80g			

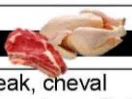

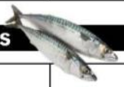

3

	ALIMENTS	FREQUENCE							QUANTITE			
		Jamais ces 4 dernières semaines	1 x par mois	2 à 3 x par mois	1 à 2 x par semaine	3 à 4 x par semaine	1 x par jour	2 x ou plus par jour	Portion de référence	Votre portion moins	Votre portion pareil	Votre portion plus
**Légumes**												
30	haricots verts, épinards								180g = 1 gobelet yaourt			
31	choux-fleurs, brocolis								180g = 1 gobelet yaourt			
32	tomates								180g = 1 grosse ou 2 petites tomates			
33	carottes								100g = 1 gobelet yaourt			
34	salade verte								100g = 1 gobelet yaourt			
35	vinaigrette								15g = 1 cuill. à soupe			
36	petits pois, maïs en grains								75g = ½ gobelet yaourt			
37	avocat								100g = ½ avocat			
38	soupe de légumes claire (bouillon de légumes)								300ml= 1 bol ou 1 assiette à soupe			
39	soupe de légumes épaisse (aux pois, aux haricots, minestrone)								300ml= 1 bol ou 1 assiette à soupe			
40	pommes de terre nature								150g= 1 grosse ou 2 petites p. de terre			
41	frites								120g = 1 poignée			

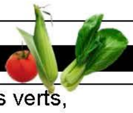

4

ALIMENTS		FREQUENCE							QUANTITE			
		Jamais ces 4 dernières semaines	1 x par mois	2 à 3 x par mois	1 à 2 x par semaine	3 à 4 x par semaine	1 x par jour	2 x ou plus par jour	Portion de référence	Votre portion: moins	pareil	plus
**Autres**												
42	pâtes alimentaires								180g cuites = 70g crues			
43	raviolis, tortellinis, cannellonis								150g cuits = env. 10 raviolis			
44	riz								150g cuit = 60g cru			
45	sauce tomate								80g = 2 cuill. à soupe			
46	semoule de blé, couscous								80g cuit = 2 cuill. à soupe			
47	pizza								1/2 pizza de la pizzeria			
48	quiche lorraine								100 g = 1 ramequin			
49	œufs								2			
50	tofu								65g			
**Vous ajoutez sur vos plats ou sur votre pain?** 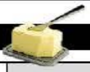												
51	margarine allégée								10 g = 1 cuill. à café			
52	beurre								10g = portion bistrot			
53	crème fraîche entière 35%								20g= 1 cuill. soupe			
54	mayonnaise								10 g = 1 cuill. à café			
**Fruits** 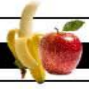												
55	banane, pomme, poire, prune, raisin								150g = 1 fruit ou 1 petite grappe			
56	orange, mandarine, clémentine								150g = taille d'une orange			
57	pêche, nectarine, abricot, melon								150g = taille d'une pêche			
58	fraises, framboises, myrtilles, cassis								120g = 1 petite barquette			
59	kiwi								80g = 1 fruit			
60	fruits en conserve, compote sucrée								150g = taille du gobelet de yaourt			

5

ALIMENTS		FREQUENCE							QUANTITE			
		Jamais ces 4 dernières semaines	1 x par mois	2 à 3 x par mois	1 à 2 x par semaine	3 à 4 x par semaine	1 x par jour	2 x ou plus par jour	Portion de référence	Votre portion: moins	pareil	plus
**Pâtisseries, Sucreries** 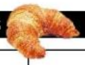												
61	croissant, pain au chocolat								1 pièce			
62	tarte aux fruits								120g = 1 tranche			
63	gâteau à la crème								90g = 1 gâteau			
64	cake, pâtisserie sèche								90g= 1 grosse tranche			
65	biscuits secs, cookies								20g= 3 biscuits			
66	chocolat								15g= 3 carrés			
67	confiture, miel								20g = 1 cuill. à soupe			
68	glace, sorbet								100g = 1 boule			
69	sucre artificiel (assugrin, aspartam)								1 comprimé			
70	sucre								1 morceau et ½			
**Pour cuisiner** 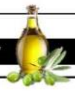												
71	beurre								10g = portion bistrot			
72	margarine								10g = 1 cuill. à café			
73	huile d'olive								2 cuill. à café			
74	huile d'arachide								2 cuill. à café			
75	huile de tournesol								2 cuill. à café			
**Vitamines, suppléments alimentaires**												
76	vitamine C								500 mg			
77	vitamine E								1'000 mg			
78	multivitamines								1 comprimé			
79	son								1 cuill. à soupe			
80	comprimé d'ail								1 comprimé			

6

ALIMENTS		FREQUENCE							QUANTITE			
		Jamais ces 4 dernières semaines	1 x par mois	2 à 3 x par mois	1 à 2 x par semaine	3 à 4 x par semaine	1 x par jour	2 x ou plus par jour	Portion de référence	Votre portion moins	pareil	plus
**Boissons**												
81	café								1 tasse			
82	lait écrémé (à 0%) dans le café								2 cuill. à soupe			
83	lait entier ou mi-écrémé dans le café								2 cuill. à soupe			
84	crème à café								1 portion de bistrot			
85	lait écrémé (à 0%) à boire								2dl = 1 verre			
86	lait entier ou mi-écrémé à boire								2dl = 1 verre			
87	Aproz, Valser, San Pellegrino, Passuger								2dl = 1 verre			
88	Perrier, Vittel, Volvic								2dl = 1 verre			
89	eau du robinet, Henniez, Evian, Vichy								2dl = 1 verre			
90	limonade, coca, soda, sirop de fruits								2dl = 1 verre			
91	jus de fruits frais								2dl = 1 verre			
92	jus de fruits en bouteille ou en brique								2dl = 1 verre			
93	thé, infusion								2dl = 1 tasse			
94	bière								3dl = 1 cannette			
95	vin, champagne								1dl et ½ = 1 verre à vin			
96	apéritifs type anisette, Martini								½ dl = ¼ de verre			
97	alcool fort type whisky, eau de vie, liqueur								1dl = ½ verre			

7

	ALIMENTS	FREQUENCE							QUANTITE			
		Jamais ces 4 dernières semaines	1 x par mois	2 à 3 x par mois	1 à 2 x par semaine	3 à 4 x par semaine	1 x par jour	2 x ou plus par jour	Portion de référence	Votre portion moins	Votre portion pareil	Votre portion plus
**Autres**												
98	oignons								1 pièce			
99	concombres, courgettes, aubergines								150g = 1 gobelet yaourt			
100	poivrons								½ pièce			
101	champignons								150g = 1 gobelet yaourt			
102	autres huiles, par ex. huile de colza								2 cuill. à café			
103	sauces grasses, par ex. sauce à la crème, sauce avec du lait de coco								½ dl = 3 cuill. à soupe			
104	sauce bolognaise								160g = 4 cuill. à soupe			
105	fruits à coque (amandes, noix, cacahuètes, noisettes etc.)								1 poignée			
106	sel ajouté								1 pincée			
107	chips								1 poignée			
108	olives noires et vertes								5 pièces			
109	autres fromages, (Emmental, fromage à raclette etc.)								40g = taille d'une boîte d'allumettes			
110	chocolat chaud ou froid, boisson mélangée à base de lait, shake de protéines								2dl = 1 verre			
111	Nutella								20g = 1 cuill. à soupe			
112	birchermüesli avec yaourt								180g = 1 gobelet yaourt			

	ALIMENTS	FREQUENCE							QUANTITE			
		Jamais ces 4 dernières semaines	1 x par mois	2 à 3 x par mois	1 à 2 x par semaine	3 à 4 x par semaine	1 x par jour	2 x ou plus par jour	Portion de référence	Votre portion		
										moins	pareil	plus

S'il manque des aliments que vous avez mangés le mois dernier, décrivez-les-ci-dessous

113												
114												
115												
116												
117												
118												

Sur cette page vous trouvez de la place pour partager avec nous vos questions, remarques, critiques et suggestions à propos de l'étude. Nous vous sommes très reconnaissants pour vos commentaires.

9

Supplemental Figure S2. Food frequency questionnaire in French used in the Swiss Childhood Cancer Survivor Study (SCCSS)-Nutrition study
